# *Aspergillus nomiae* and *fumigatus* Ameliorating the Hypoxic Stress Induced by Waterlogging through Ethylene Metabolism in *Zea mays* L.

**DOI:** 10.3390/microorganisms11082025

**Published:** 2023-08-07

**Authors:** Khalil Ur Rahman, Kashmala Ali, Mamoona Rauf, Muhammad Arif

**Affiliations:** 1 Department of Biotechnology, Abdul Wali Khan University Mardan, Mardan 23200, Pakistan; khalilurrahman@awkum.edu.pk; 2 Department of Botany, Abdul Wali Khan University Mardan, Mardan 23200, Pakistan; kashmalaali92@gmail.com

**Keywords:** ACC deaminase enzyme, endophytic fungus, ethylene, antioxidants, waterlogging, hypoxia

## Abstract

Transient and prolonged waterlogging stress (WS) stimulates ethylene (ET) generation in plants, but their reprogramming is critical in determining the plants’ fate under WS, which can be combated by the application of symbiotically associated beneficial microbes that induce resistance to WS. The present research was rationalized to explore the potential of the newly isolated 1-aminocyclopropane-1-carboxylic acid (ACC) deaminase-producing fungal endophytic consortium of *Aspergillus nomiae* (MA1) and *Aspergillus fumigatus* (MA4) on maize growth promotion under WS. MA1 and MA4 were isolated from the seeds of *Moringa oleifera* L., which ably produced a sufficient amount of IAA, proline, phenols, and flavonoids. MA1 and MA4 proficiently colonized the root zone of maize (*Zea mays* L.). The symbiotic association of MA1 and MA4 promoted the growth response of maize compared with the non-inoculated plants under WS stress. Moreover, MA1- and MA4-inoculated maize plants enhanced the production of total soluble protein, sugar, lipids, phenolics, and flavonoids, with a reduction in proline content and H_2_O_2_ production. MA1- and MA4-inoculated maize plants showed an increase in the DPPH activity and antioxidant enzyme activities of CAT and POD, along with an increased level of hormonal content (GA_3_ and IAA) and decreased ABA and ACC contents. Optimal stomatal activity in leaf tissue and adventitious root formation at the root/stem junction was increased in MA1- and MA4-inoculated maize plants, with reduced lysigenous aerenchyma formation, ratio of cortex-to-stele, water-filled cells, and cell gaps within roots; increased tight and round cells; and intact cortical cells without damage. MA1 and MA4 induced a reduction in deformed mesophyll cells, and deteriorated epidermal and vascular bundle cells, as well as swollen metaxylem, phloem, pith, and cortical area, in maize plants under WS compared with control. Moreover, the transcript abundance of ethylene-responsive gene *ZmEREB180*, responsible for the induction of the WS tolerance in maize, showed optimally reduced expression sufficient for induction in WS tolerance, in MA1- and MA4-inoculated maize plants under WS compared with the non-inoculated control. The existing research supported the use of MA1 and MA4 isolates for establishing the bipartite mutualistic symbiosis in maize to assuage the adverse effects of WS by optimizing ethylene production.

## 1. Introduction

Soil flooding or waterlogging (WL) is the result of an excessive buildup of water in the soil brought on by prolonged periods of heavy precipitation, poor drainage, excessive irrigation, and flash floods [1]. Waterlogging harms almost 10% of the world’s land. Floods were accountable for two-thirds of all crop losses and damage worldwide between 2006 and 2016. Waterlogging is attributed to low light, deficient gaseous exchange, hypoxia, and anoxia, and it covers the roots of plants. By 10,000 times lessening O_2_ diffusion than air, it inhibits aerobic activity, including soil root respiration. Reactive oxygen species (ROS) are produced when the ETC of chloroplasts and mitochondria is inhibited by the anoxic condition [2]. WL causes crop root hypoxia, the inhibition of root respiration, stomatal conductance, reduced CO_2_ entry, decreased transpiration rate, decreased photosynthetic rate, and, ultimately, decreased or failed crop yield [3]. Hypoxic and anoxic soils inhibit several biological processes, including root water uptake, phytohormone interactions, ionic uptake and transport, and antioxidant activities. Additionally, WL results in cascading changes in the physicochemical properties of the soil, which can effectively increase ionic and elemental toxicity, cause additional nutrient loss through leaching or the release of greenhouse gases, and/or increase plant phenolic and volatile fatty acid levels [4]. Plants can, however, adapt to WL by changing their morphology, architecture, biochemical responses, and metabolism. Through amplified catalase (CAT), ascorbate peroxidase (APX), and superoxide dismutase (SOD) activities that scavenge the ROS produced by plant cells induced by WL, plant systems have adapted to rely on antioxidant potential for the sustainability of the dynamic balancing of ROS under WL-induced redox stress [5,6]. Superoxide anion radicals (O^2−^) and hydrogen peroxide (H_2_O_2_) production, abnormal membrane homeostasis, malondialdehyde (MDA) accumulation, and the acceleration of leaf senescence are all caused by the overproduction and formation of ROS that WL causes in plants [7]. Supplementing exogenous regulators is one of the most popular methods for increasing the antioxidant potential of crops under waterlogged stress. Aminobutyric acid, for instance, increased the activity of antioxidant enzymes that reduced MDA content and H_2_O_2_ to improve the ability of maize (*Zea mays* L.) to withstand WL stress [8]. Plants can also adapt to stress by being exposed to H_2_O_2_ in low concentrations. The formation of aerenchymatous adventitious roots as the superficial root system, which is controlled by phytohormones (auxin, ethylene, abscisic acid, jasmonates, cytokinin, and gibberellin), is one of the primary morphological, as well as anatomical, adaptations [9]. To reduce WL stress by supplying O_2_ to the root tip, rice roots are recognized as developing lysigenous aerenchyma and a barrier to radial O_2_ loss [10]. Despite significant efforts made in recent years, the molecular mechanisms underlying these physiological and morphological responses are still not fully understood. The molecular basis for these morphological adaptations is RNA dynamics driven by miRNA expression modulation, according to earlier reports in this venture. Through hormonal signal transduction, *miRNAs* assist and regulate the equilibrium amid the response to flooding and waterlogging stress (WS) and plant development. The phytohormones ethylene, GA_3_, ABA, and auxin signal transduction pathways are the primary elements connecting the intricate network of responses to flooding, and they work in concert to confer plant adaptive responses to flooding constraints [11]. Several *miRNAs* that are flooding-responsive regulate target mRNAs, which encode transcription factor genes involved in hormonal metabolism and signaling pathways, orchestrating changes in morphology in the various plant tissues as a result of flooding [11,12]. Additionally, genetic engineering has been used to investigate the molecular basis of WL tolerance in some plant species, including *Arabidopsis*, maize, cucumber, and cotton [13]. For instance, the *Actinidia deliciosa pyruvate decarboxylase 1 (AdPDC1)* gene from Kiwi fruit was overexpressed in transgenic *Arabidopsis thaliana* and improved WL tolerance [13].

WL has also been demonstrated to affect the gene expression of the metabolic enzymes (*cinnamoyl-CoA reductase 2*, *phenylalanine ammonia-lyase 6*, and *ferulate 5-hydroxylase 2*), along with induction of gene expression controlling the growth and stress-related hormonal metabolism, such as IAA biosynthesis and transport (*YUC1*, *TDC*, *PIN9)*, ethylene biosynthesis (*ACO2*, *ACS7*), Jasmonic acid metabolism (*AOS1*, *LOX8*, *AOC1*, *JAR1*), and Gibberellic acid metabolism (*GA2ox8* and *GA3ox2),* cytokinin metabolism (*IPT5-2*, *LOG1, CKX5*, and *ZOG2*), as well as ABA biosynthesis *(NCED1)* [14,15]. Additionally, anoxia reduces the abundance of the denitrification gene *nirS* in the rhizosphere of wheat [16]. Several proteins were also differentially expressed in response to WL stress in tomato leaves and adventitious roots of cucumbers [17]. Previous studies have demonstrated that omics (Genomics, Transcriptomics, Proteomics, and Metabolomics) are effective tools for engineering, regulating gene expression, and post-transcriptional and translational modifications to drive plant stress tolerance responses. Nevertheless, more research needs to be conducted on the molecular basis of WL tolerance. Furthermore, efforts have been made to create WL-tolerant varieties using the conventional quantitative trait loci (QTL) strategy and classical genomics; however, less has been investigated for exploring the underlying molecular mechanism of waterlogging tolerance due to time-consuming field trials in challenging environments (WTs). Recently, scientists from around the globe began utilizing growth-promoting endophytic fungi from exotic environments, which has been proven to be a successful biological tactic for increasing plant stress tolerance (biotic and abiotic, by promoting ecological adaptability to harsh environments, for example, in rice, luffa, moringa, wheat, tomato, and okra [18,19,20,21,22,23,24,25]. Nevertheless, the WL tolerance response and mechanism in maize induced by certain plant–fungal interactions have not yet been studied. Endophytic fungi have been shown to generate a variety of metabolites, hormones, antioxidants, and enzymatic proteins, such as ACC deaminase, which improves wheat development and enhances plant tolerance to NaCl stress and is produced by *Trichoderma longibrachiatum T6* (TL-6) [26]. According to [27], *T. asperellum* T203 production of ACC deaminase controlled the level of endogenously produced ACC, enhanced elongation of canola roots, and boosted resilience to abiotic stress. Ref. [28] also isolated and identified the ACC deaminase producing plant growth promoting microbes from rice rhizosphere the evaluated the potential for induction of salt stress tolerance in rice. Because ET precursor ACC may be catabolized to a-ketobutyrate and ammonia by fungal endophytes expressing the ACC deaminase gene, it is thought that ACC deaminase-producing endophytic fungal association with plants controls ethylene (ET) [29]. ET, the smallest phytohormone (C_2_H_4_), influences both the salicylic acid and jasmonic acid pathways and is involved in fruit ripening, seed germination, plant senescence, and immunity [30]. Multiple functions of ET as a signaling molecule facilitate several biochemical and molecular pathways working between ET and stress-induced growth. Submergence, heat, shade, heavy metal, high salt exposure, limited food availability, and water scarcity are all abiotic stressors that cause ET synthesis [31]. As a result, the development of root colonization and chemical communication by ACC deaminase-producing fungal endophytes have a major impact on plant physiology by changing ET-related cell signaling and gene expression modification to improve overall plant performance. ET production over its threshold level, on the other hand, works as “stress ethylene”, which is intimidating in terms of plant growth and development. The effects of stress ethylene in plants have been alleviated by certain plant-associated bacteria that possess the enzyme 1-aminocyclopropane-1-carboxylate deaminase [32]. This feature of assigning resistance to abiotic stressors via ACC deaminase enzyme activity and certain additional ways of plant growth-promoting microorganisms alleviating stresses in host plants is known as “induced systemic tolerance” [33]. Hence, plant growth-promoting fungal endophytes with ACC deaminase enzyme activity are critical in mitigating the negative consequences of WL stress. There have only been a few papers that illustrate the involvement of endophytic fungus in improving WS-induced hypoxic stress tolerance in plants. *Aspergillus fumigatus* SG-17 endophyte from *Myricaria flaxiflora* (flood-tolerant plants) showed that the styrene antioxidant (Z)-N-(4-hydroxystyryl) formamide (NFA) may efficiently reduce flooding stress in *Arabidopsis thaliana* [34]. The relationship between ACC deaminase-generating endophytic fungus and the maize plant for WL stress resistance via ethylene bio-reduction has never been studied before. As a result, the current study was rationalized to investigate the potential of ACC deaminase-producing endophytic fungi for alleviating WL stress in maize.

## 2. Methods

### 2.1. Isolation of Endophytic Fungi from Seeds of Moringa oleifera *L.*

The growth-promoting endophytic fungi were isolated from the seeds of *Moringa oleifera* L. collected from the field area of the city of Lahore (31°15′–31°45′ north latitudes and 74°01′–74°39′ east longitudes), built near the Ravi River of Pakistan. Pods were opened wide to recruit the seeds and surface sterilized to eliminate the non-endophytic microorganisms, which is essential for precisely exploring the endophytes. Afterward, the diversity of fungal endophytes is relatively stable at higher concentrations of NaClO. To achieve endophytic fungal isolates, the seeds were first surface sterilized by using ethanol (70% for 1 min) and NaClO (2.0% for 2 min), followed by washing with double-distilled H_2_O. Sterilized seed tissues excised with a sharp razor were incubated on Hagem minimal medium (25 °C, one week). The colonies that emerged directly from the seed tissues were subjected to re-culturing on potato dextrose agar medium (25 °C, one week), and purified fungal isolates were upscaled using Czapek culture broth liquid media (30 °C, 120 rpm, seven days). A pellet of fungal biomass and supernatant were separated and stored at −70 °C to conduct biochemical and metabolic analysis.

### 2.2. ITS Sequencing of Fungal Isolates for Molecular Identification

Whole genomic DNA was extracted using the DNeasy plant micro kit (QIAGEN, Valencia, CA, USA), as described by [35]. Endophytic fungi were identified by sequencing the *ITS1-5.8S-ITS2* region of *18S rDNA* with unique primers *ITS1 (5-TCCGTAGGTGAACCTGCGG-3′)* and *ITS4 (5′-GCTGCGTTCTTCATCGATGC-3′),* as previously described [36], and closely related sequences were finally extracted and aligned using CLUSTAL W. For performing the phylogenetic and molecular evolutionary analyses MEGA version ‘X’ software was used [37].

### 2.3. Macro- and Microscopic Phenotyping of Seed Endophytic Fungal Isolates

The deceptive morphological features of endophytic fungi were evaluated according to standard protocols for light microscopy by visualizing under the light microscope (Binocular NSL-CX23 Olympus, Tokyo, Japan) at the magnifications 40× and 100×. Staining solution (Lactophenol cotton blue) was used for the microscopic evaluation of the specified fungal components, as described earlier [38].

### 2.4. Metabolic Analysis of Seed Endophytic Fungal Isolates

Evaluation of auxins (IAA) produced by MA1 and MA4 isolates was carried out by Salkowski assay through a spectrophotometer at 535 nm, as mentioned earlier [39]. The spore suspension (1 mL) of MA1 and MA4 (1 × 10^8^ spores mL^−1^) was inoculated to the 50 mL of Czapek media (MgSO_4_·7H_2_O 0.05%, peptone 1%, C_6_H_12_O_6_ 1%, KCl 0.05%, FeSO_4_·7H_2_O 0.001%, pH 7.3) using 100 mL conical flasks and kept at 30 °C with 120 rpm in a shaking incubator for 7 days. After centrifugation at 3000 rpm for 20 min, the supernatants were filtered through a 0.45 m cellulose acetate filter (DISMIC^®^; Frisenette ApS, Knebel, Denmark) to obtain the fungal culture filtrate (FCF), which was acidified to pH 2.8 with 1 N HCl and extracted three times with 20 mL ethyl acetate. The ethyl acetate fractions after combining were subjected to evaporation using a rotary evaporator, under vacuum, at 45 °C. The residue was resuspended in 50% methanol (3 mL). Then, 1 mL of supernatant was mixed with 2 mL of Salkowski reagent and kept in the dark for 30 min. The resultant reddish color was read after 30 min at 535 nm in a spectrophotometer (UV/VIZ, PerkinElmer Inc., Waltham, MA, USA). Salkowski reagent (2 mL) was used as a blank, mixed with 1 mL of Czapek medium. Various known concentrations (0.5–1000 µg/mL) of IAA (Sigma Aldrich, Burlington, MA, USA) were prepared for the attainment of the calibration curve.

The proline content was quantified as mentioned earlier [40], by mixing 0.1 mL of FCF of MA1 and MA4 isolates with 4 mL of 3% sulfosalicylic acid and centrifuged at 3000 rpm for 5 min, and 2 mL of acid ninhydrin was added to the 2 mL of sub permanent. The samples were heated for 1 h in a water bath, and 4 mL of toluene was added. The toluene layer was separated, and the absorbance was noted at 520 nm. The plant tissues were also processed and analyzed similarly.

Total flavonoids were quantified, as described earlier [41], by mixing the 0.5 mL of FCF of MA1 and MA4 isolates with 0.1 mL of potassium acetate, 0.1 mL of AlCl_3_ (10%), and 4.3 mL of 80% methanol, and then vortexed thoroughly. The reaction mixture was incubated at room temperature in the dark for 2 h. Absorbance was at 415 nm. The blank consists of 2 mL of reaction buffer (0.1 mL AlCl_3_ + 0.1 mL potassium acetate, and 4.3 mL methanol).

Total phenolic content was determined using the method described earlier [41]. To this end, 10 mL of methanol was added to the 1 mL of FCF of MA1 and MA4 isolates, followed by mixing and centrifuging. Next, 0.5 mL of Folin–Ciocâlteu reagent (50%) + 2 mL of Na_2_CO_3_ (20%) was added to the centrifuge sample extract (5 mL). The mixture was heated for 1 min, until blue coloration appeared due to the complex redox reaction of phenolics with the phosphomolibidic acid in alkaline Folin–Ciocâlteu reagent, resulting in a molybdenum blue complex. Finally, the absorbance of the reaction was measured at 650 nm, spectroscopically.

### 2.5. Assessment of the Antagonistic Activity by the Dual-Culture Plant Method

A dual-culture experiment was conducted to evaluate the antagonistic activity of MA1 and MA4 isolates against each other through co-cultivation test on PDA nutrient medium plate. A total of 20 μL of MA1 and MA4 each, at 1 × 10^7^ spores mL^−1^, were inoculated in the center of PDA plates. The control cultures were contained only MA1 and MA4. The experiment was carried out in triplicate and incubated at 26 ± 2 °C. Observation showed that MA1 and MA4 completely covered each other, without any growth suppression or inhibition. MA1 and MA4 isolates showed compatible growth with each other.

### 2.6. Induction and Evaluation for ACC Deaminase Enzymatic Activity

The evaluation of ACC deaminase activity was performed by determining the production of a-ketobutyrate through the deamination reaction of ACC, which was expressed as μmol a-ketobutyrate mg^−1^ protein h^−1^. For the determination of the ACC deaminase activity, the spore suspension of MA1 and MA4 (1 × 10^8^ spores mL^−1^, 1 mL) was inoculated in czepak media (60 mL) supplemented with 0.5 to 2 mM ACC (the control was without ACC supplementation), and the culture was kept in a shaker incubator (28 °C, 180 rpm min^−1^, 5 days). The fungal mycelia were harvested and homogenized with 2.5 mL of Tris buffer (0.1 M, pH 8.5), followed by the addition of toluene (25 μL) to the 200 μL aliquot. The mixture was vortexed and incubated (30 °C, 15 min), followed by an addition of 1 mL of HCl (0.56 N), then centrifuged (3000 rpm, 10 min). Subsequently, the supernatant was supplemented with 800 μL of HCl (0.56 N), 300 μL of 2, 4-dinitrophenylhydrazine, and 2 mL of NaOH (2 N), followed by an incubation period (30 °C, 30 min). The quantification of ACC deaminase enzymatic activity was performed spectrophotometrically, assessing the α-ketobutyrate production at 540 nm, as described earlier [26,42], using ketobutyrate (0.5–500 μmol) as a standard.

### 2.7. Induction and Evaluation for ACC Deaminase Gene Expression Analysis by RT-qPCR

Gene expression for the ACC deaminase enzyme was measured using real-time quantitative PCR (RT-qPCR), as explained previously [18,27]. Isolation of total RNA from each fungal biomass sample was followed by a DNA digestion procedure through DNAase supplementation and purification through RNeasy Mini columns (Qiagen, Hilden, Germany). An equal amount of purified RNA (2 µg) was obtained as the template for the first strand of cDNA synthesis using SuperScript II reverse transcriptase (Invitrogen, Lyon, France). The primer oligo (dT) was used for cDNA synthesis. Finally, the SYBR Green master mix (Applied Biosystems Applera, Darmstadt, Germany) was used to perform RT-qPCR on the ABI PRISM 7900HT (Applied Biosystems Applera, Darmstadt, Germany).

The 1-aminocyclopropane-1-carboxylate deaminase enzyme gene was amplified from *Aspergillus funmigatus* (*AFUA_2G01030*) using forward primer (*F-TGGGGGTGTTCTTCGATGTG*) and reverse primer (*R-ATCAATCCCGATAACCCGCC*)*,* and from *Aspergillus nomius NRRL 13137* (*XM_015553204*) with forward primer (*F-AACTTAAACCCCGCCACCAT*) and reverse primer (*R-AATCTCCGGGAGGAGTGTGA*). ACC deaminase enzyme gene primers were designed through primer 3.0 [43], and ACC deaminase enzyme transcript abundances were normalized to *actin* (*actA*) (*AFUA_6G04740*) as the internal control reference gene, earlier reported earlier [44], and *calmodulin* (*CAL*) (*AY974341*) as the reference gene with primers *CAL-F-CAAGGAGTTGGGCACTGTCA* and *CAL-R-CCATTGTTGTCGGCGTCAAC*, as reported earlier [45].

### 2.8. Root Length Elongation Bioassay with Ethylene Response Mutants

Etiolated *Arabidopsis* seedlings overproduce ethylene and exhibit a morphological growth defect known as the triple response, specified by the shortening and radial swelling of the hypocotyl, inhibition of root elongation, and exaggeration of the curvature of the apical hook. *Ethylene overproducers (eto1 mutants)* have previously been identified and characterized earlier [46]. Mutations in *eto1*, which is a negative regulator of an *ACS5* gene (encoding ACC synthase, the first step in the pathway), induced ethylene overproduction by overproduction of ACC precursor and were utilized to screen and evaluate the response of ACC deaminase enzyme-producing fungal isolates on the ethylene production response of *eto1* mutants.

The seeds from *Arabidopsis thaliana* Col-0 wild type and *ethylene overproducer (eto1)* mutant were surface sterilized (1 min in 70% ethanol, 30 min in 20% sodium hypochlorite). After imbibition and subsequent stratification (4 °C for 4 days), the seeds were incubated (dark condition, 23 °C, 66 to 72 h) for germination on the liquid MS medium (½ strength, sucrose 1%) [47], in glass petri plates embedded with sterilized filter papers. A fungal spore suspension culture (1 × 10^7^ spores mL^−1^) from MA1 and MA4 was inoculated into the liquid MS medium. Control seeds were incubated without a fungal culture inoculum. Twenty seeds were placed in each glass petri dish filled with 15 mL of MS medium, with three layers of autoclaved filter papers as embedding support, and sealed properly. Six replicate plates were used for each treatment. The assay was repeated three independent times. After 3 days, the hypocotyle root length was measured. Visual inspections of phenotypic modifications enabled quick evaluation of ET-related morphological changes that arose during the growth of etiolated *eto1* seedlings. The hypocotyl growth kinetics of etiolated *eto1* were measured with and without the inoculation of endophytic fungal cultures to assess the reversal of the triple response induced by ethylene biosynthesis and metabolism. Col-0 etiolated seedlings were used as the wild-type control.

The experiment was comprised of four treatments, individually for Col-0 (wild-type) and *eto1* mutant seedlings, as listed below.

1. Col-0 = Control (CK)
2. Col-0 *=* Inoculated with MA1
3. Col-0 *=* Inoculated with MA4
4. Col-0 = Inoculated with MA1 and MA4
5. *eto1* = Control (CK)
6. *eto1 =* Inoculated with MA1
7. *eto1 =* Inoculated with MA4
8. *eto1* = Inoculated with MA1 and MA4

### 2.9. Maize-Endophytic Fungal Association Bioassay

#### 2.9.1. Experimental Design for Plant Bioassay

*Zea mays* (Var. Gulibathi) seeds were received from the Agricultural Research Institute (ARI), Ternab, Peshawar. Healthy seeds were washed three times with autoclaved distilled water, followed by sterilization with ethanol (70%) and washing with distilled water three times. Seeds were then sown in soil pre-mixed with fungal biomass (3 g/500 g of soil). The same quantity of irrigation water was used to irrigate the controls. The experimental setup included 12 plastic pots or treatments (8.5 cm in diameter, 12.5 cm in depth), and 500 g of sterile sandy loam soil, which was analyzed for physiochemical properties, as shown in Table 1.

In all pots, the soil water content was adjusted after sowing and kept at 80% of the field capacity. Throughout the six-week experiment period, pots were watered every morning to keep the soil as close to an 80% field capacity as possible. This was accomplished by weighing and adjusting the water in ten randomly chosen pots from each experiment. All pots were weighed and watered equally to further optimize the irrigation regimen and minimize the effects of watering on plant growth.

Seed pots were kept in a growth chamber (day/night cycle: 14 h/28 °C ± 0.3; 10 h/25 °C ± 0.3; relative humidity 70%). The experiment was designed with a completely randomized design (CRD) with 8 treatments and 12 technical replicates each, and the experiment was repeated in 3 biological replicates. Each treatment contained 10 pots, each measuring 6 to 8 cm in height and diameter, each containing 4 seedlings (for a total of 120 seedlings per each treatment), irrigated with 4 mL of water in each pot during the whole experiment, and incubated in a controlled environment (300 μmol m^−2^ s^−1^ light intensity, 25–28/15–17 °C temperature (day/night), 70/85% relative humidity, 17/7 h photoperiod).

#### 2.9.2. Colonization Frequency

According to [48], the calculation for the colonization frequency (%CF) of MA1 and MA4 endophytic fungi was performed using the formula %CF = (Ncol/Nt)/100, where Ncol is the number of segments colonized by each fungus, and Nt is the total number of segments studied.

#### 2.9.3. Waterlogging Treatment

Plants (15 days old) were subjected to waterlogging (WL) stress for 7 days straight, followed by a 7-day recovery period. The pots (8 cm in height) were placed in plastic tanks (40 cm *×* 20 cm *×* 8 cm, length: width: height) and subjected to flooding with tap water by filling the tanks to a height of 7.8 cm, ensuring the upper level of the water always stayed below the pot’s top edge, and plants were kept in the tanks without being submerged for untreated control.

There are eight treatments in this experiment:

1. Control (CK) = no waterlogging stress
2. MA1 = endophyte inoculated
3. MA4 = endophyte inoculated
4. MA1 and MA4 = consortium of endophytes inoculated
5. WL = waterlogging stress
6. WL + MA1 = endophyte inoculated
7. WL + MA4 = endophyte inoculated
8. WL + MA1 and MA4 = waterlogging stress and endophyte inoculation

Phenotypic observations were collected at four main time points:

1. 0 days of waterlogging treatment and 0 days of endophytic inoculation (8 DAG)
2. 0 days of waterlogging treatment and 8 days of endophytic inoculation (15 DAG)
3. 7 days of waterlogging treatment and 15 days of endophytic inoculation (22 DAG)
4. 7 days after waterlogging treatment (recovery period) and 20 days of endophytic inoculation (29 DAG)

Morphological, biochemical, and physiological analyses were carried out 7 days after the recovery period (29 DAG).

#### 2.9.4. Morphological and Physiological Analysis of Maize Seedlings

Various growth characteristics, including shoot/root length, shoot/root dry weight, and water retention, were assessed after the seedlings were harvested. Fresh weight measurements exhibit variations because of environmental and technical issues, including temperature, relative humidity, air currents, the size of the experiment, and blotting excess moisture from tissues during harvest and/or preparation of tissues for weighing. To assess the effects of growth-promoting endophytic fungi on maize plants, the dry weight and water content were measured as important parameters, according to the procedure described earlier [49]. Maize roots were carefully washed with tap water after being cut apart from the shoot tissues to prevent root rot and remove extra soil. An ambient temperature of 21 ± 2 °C was used to weigh fresh shoots and roots on a scale (0.01 g). Extra moisture on the roots was blotted with brown paper towels before weighing the fresh weight of the roots. The samples were then dried in an oven at 70 °C for 72 h to achieve a constant weight. A scale was used to weigh dried shoots and roots (±0.01 g), and the water content of the shoot and root was calculated.

#### 2.9.5. Determination of Chlorophyll Content

For the estimation of chlorophyll content, the method described earlier [50], was used by taking around 0.1 g of tissue from fresh leaves homogenized with 3 mL of 80% acetone, centrifuged (1000 rpm; 5 min; room temperature), and adjusting the final volume up to 7 mL with 80% acetone. Absorbance was recorded at 663, 645, 480, and 510 nm.

#### 2.9.6. Stem and Stomatal Anatomical Evaluation

The stomatal and stem anatomy was assessed as described earlier [19]. Peels were taken from the abaxial surface of leaves and floated on distilled water for two hours, while being continuously illuminated to study stomatal anatomy. The peels were then submerged in distilled water with a pH of 5.5, and the stomata were examined under a 100× light microscope (Binocular NSL-CX23 Olympus, Tokyo, Japan). Digital cameras were used to capture stomatal images (Canon, Tokyo, Japan). The stomatal aperture was calculated with ImageJ (http://fiji.sc/, accessed on 12 January 2022), and calibration was performed using a µm ruler. By applying a free-hand sectioning technique, stem transverse sections were cut with a razor blade and subjected to staining with safranin staining solution and subsequent visualization under a light microscope at a magnification of 40×.

#### 2.9.7. Visualization and Quantification of H_2_O_2_

For H_2_O_2_ quantification, the potassium iodide (KI) protocol described earlier [51], was used by taking 0.5 g of finely crushed leaf tissue samples homogenized with 3 mL TCA and centrifuging through 12,000 rpm for 15 min and vertex. The 0.5 mL supernatant was extracted and mixed with an equal volume of 10 mM potassium phosphate buffer, then 1 mL potassium iodide. The reaction mixture was used to record the absorbance at 390 nm. The phosphate buffer alone was used as a blank.

The method described earlier [52], was followed for the histochemical staining with 3,3-diaminobenzidine (DAB) to monitor H_2_O_2_ production and a freshly prepared DAB solution. After being incubated for 12 h under vacuum with DAB staining solution (0.5 mg/mL), segments of the fully expanded, third leaf from 27-day-old plants had their chlorophyll removed by being placed in 90% ethanol at 70 °C for 10 min. The DAB polymerization caused H_2_O_2_ to appear brown. A digital camera (Canon) was used for the photography of the leaf segments.

#### 2.9.8. Extraction and Quantification of Antioxidant Enzymes

First, 0.2 mL of leaf extracts was combined with 600 microliters of PBS (50 mM, pH 7.0), 100 microliters of ascorbic acid (0.5 mM), and 100 microliters of hydrogen peroxide to estimate ascorbate peroxidase using the method described earlier [53]. At a wavelength of 290 nm, the absorbance was calculated and shown as min^−1^ mg^−1^ protein.

Catalase activity was quantified through H_2_O_2_ cleavage, as described earlier [54], by taking 0.1 mL of supernatant and combining it with phosphate buffer 2.6 mL pH 7, 0.1 mM EDTA, and 400 μL of 3% H_2_O_2_. At 240 nm, the absorbance was measured.

The DPPH test was carried out as previously presented [55]. Each extract (0.2 mL) was subjected to incubation for 10 min at the ambient temperature with 2.5 mL DPPH (Sigma Aldrich, St. Louis, MO, USA) solution (0.35 mM DPPH mixed with 50% ethanol). The variations in absorbance at 517 nm were monitored, and antioxidant activity was estimated as the percentage of inhibition induced by hydrogen donor activity. Based on a standard curve developed using Trolox solution (100–1000 µM), the results were represented as µmol Trolox equivalents g^−1^ DW.

#### 2.9.9. Metabolic Analysis

The method described earlier [56], was used to assess the total soluble sugar by using phenol-sulfuric acid. A total of 0.5 g of leaf tissue of maize was homogenized with deionized water; the extract was filtered and mixed with 5% phenol and 98% sulfuric acid; the mixture remained for 1 h, and then the absorbance at 485 nm was determined by spectrophotometer.

To determine the total protein content, 0.3 g of fresh leaf tissue of maize was crushed using 1 mL of phosphate buffer and centrifuged at 3000 rpm for 10 min. Then, 0.1 mL of the supernatant was diluted with distilled water up to a volume of 1 mL, mixed with 1 mL of reagent C (reagents A and B), and stirred for 10 min. Finally, it was mixed with 1 mL of reagent D and incubated for 30 min, and the absorbance was checked at 650 nm. Folin’s reagent was used as a blank, as described earlier [57].

For the extraction of total lipids, 0.5 g of leaf powder was mixed with a 2:1 chloroform: methanol (*v*/*v*) mixture, and estimation was performed as described earlier [58].

By combining 0.5 g of leaf powder with 4 mL of 3% sulfosalicylic acid and centrifuging the mixture at 3000 rpm for 5 min, as described earlier [40], the proline content was determined. To the 2 mL of supernatant, acid ninhydrin was added. In a water bath, the samples were heated for an hour, while 4 mL of toluene was also added. After the toluene layer was cut apart, absorbance at 520 nm was measured.

#### 2.9.10. Determination of Phytohormones

Crushed leaf tissue (0.5 g) was used to estimate indole-3-acetic acid (IAA), gibberellic acid (GA_3_), and abscisic acid (ABA). For estimation of auxins (IAA) in leaf tissue, the Salkowski reagent was used, as mentioned earlier [39], by mixing 3 mL of the reagent with 0.5 g of leaf powder, mixing thoroughly, and centrifuging at 3000 rpm for 10 min, followed by incubation of the supernatant at room temperature for 20 min in the dark. The pinkish color appeared in the reaction mixture, and the absorbance was taken at 540 nm with a spectrophotometer (UV/VIZ spectrophotometer; PerkinElmer Inc., Waltham, MA, USA). Salkowski reagent (2 mL) was used as a blank, mixed with 1 mL of Czapek medium. Various known concentrations (0.5–100 g/mL) of IAA (Sigma Aldrich, Burlington, MA, USA) were prepared for the attainment of the calibration curve.

The estimation of GA_3_ and ABA contents protocol as described earlier [59], was followed by taking 0.5 g of leaf tissue powder mixed with 5 mL of reaction buffer (chloroform, methanol, and 2N NH_4_OH at a volume ratio of 12:5:3). The pH was adjusted to 2.5, and extraction was performed using ethyl acetate (15 mL), followed by adding dH_2_O (25 mL) to the reaction. The upper chloroform phase out of two phases was discarded. The aqueous phase was collected, and the pH was adjusted to 2.5. The extraction was conducted using ethyl acetate (15 mL) (three times), the reaction was incubated at 70 °C for 1 h, and the free ABA and GA_3_ content were separated. Finally, evaporation was carried out at 45 °C, and elution was performed with methanol (2 mL). The absorbance of GA_3_ was acquired at 254 nm and that of ABA at 263 nm.

#### 2.9.11. Waterlogging-Induced Marker Gene Expression Analysis in Maize

Total RNA was isolated from maize seedlings, according to the manufacturer’s instructions, by using the Gene JET Plant RNA Purification Kit (Thermo Scientifics, Waltham, MA, USA). RNase-free DNase obtained from Ambion’s TURBO DNase Kit (Cambridgeshire, UK) was applied during the extraction phase. For gene expression analysis, qPCR primers were designed using primer 3.0 [43], for WS-induced molecular marker genes, as shown in Table 2, while *ZmACTIN1* was used as an internal control, as described earlier [60]. All primers were designed by Bio Basic (BIONICS, Seoul, South Korea), and sequences with gene accession codes are shown in Table 2. *RT-qPCR* expression analysis was evaluated as described earlier (Section 2.7), using three independent biological replicates and at least three technical replicates for each sample.

### 2.10. Statistical Analysis

A complete randomized design (CRD) was adapted for all experiments. The statistical data analysis was performed using GraphPad Prism 9.0.0 (121) software, with the means and standard errors of three replicates using RM two-way analysis of variance (ANOVA). Further statistical verification was carried out using SPSS V. 21.0 (SPSS, Chicago, IL, USA) software, by applying Duncan’s multiple range test (DMRT). Finally, the significant differences were presented through different statistical bars symbolized with different letters (*p ≤* 0.05).

Calculations for the fold changes were performed as demonstrated in the formula given (mT − mC)/mC, showing the ratio of the variations among the mean of treated samples (mT) and the control samples (mC) over the mean of control samples (mC).

## 3. Results and Discussion

### 3.1. Endophytic Fungal Isolation, Identification, and Characterization

**Figure 1:**
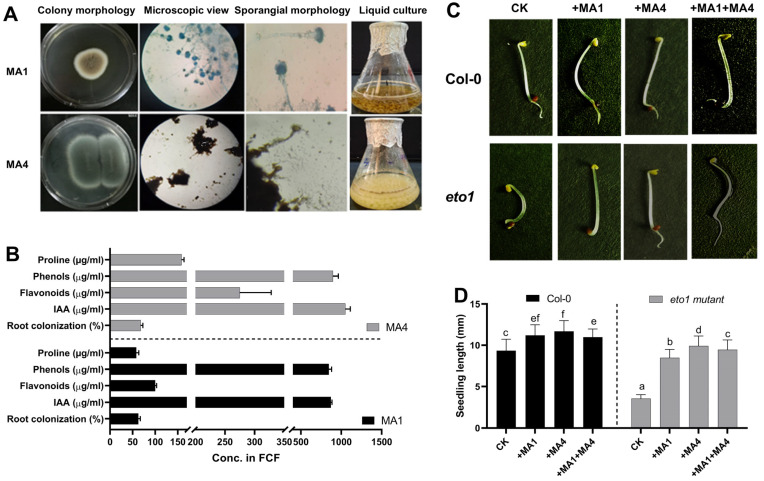
Characterization of fungal isolates. (**A**) Morphological characterization of MA1 and MA4 isolates. (**B**) Quantification of metabolites in FCF, and root colonization (%) of MA1 and MA4 with maize roots. (**C**) Influence of fungal endophytes MA1 and MA4 on the growth characteristic of *Arabidopsis thaliana eto1* mutant seedlings. (**D**) Influence of fungal endophytes MA4 on the root kinetics of *Arabidopsis thaliana eto1* mutant seedlings. Quantitative data represent means ± SE, with various letters indicating significant differences (*p* ≤ 0.05).

Fungal endophytes colonize all types of plant tissue, including seed, root, stem, and leaf, and improve the growth and defense responses of plants to mitigate biotic and abiotic stress. In the present study, a total of fifteen isolates of endophytic fungi were obtained from the seeds of *Moringa oleifera* L., and six were selected based on unique features for further investigation. Various growth parameters were tested before being administered to plant bioassays. Out of six endophytic fungal isolates, MA1 and MA4 demonstrated the highest growth response in the Czapek medium by producing the maximum biomass, which was chosen for additional investigation for plant bioassays. Based on visual characteristics, like colony texture, form, color, and growth pattern, as well as hyphae color, sporangium color, and spore morphology, the selected isolates of fungal endophytes (MA1 and MA4) were initially identified, as shown in Figure 1A.

Out of six isolates, the culture filtrate (CF) of MA1 and MA4 showed maximum biomass and growth-promoting metabolite production. MA1 grown in Czapek media had a sufficient amount of IAA (860 ± 1.7 μg·mL^−1^, proline, 61 ± 1.3 μg·mL^−1^, phenols, 863 ± 0.8 μg·mL^−1^, flavonoids, 100 ± 1.0 μg·mL^−1^ respectively). The CF of MA4 grown in Czapek media had a sufficient amount of IAA, i.e., 960 ± 1.7 μg·mL^−1^, proline, phenols, and flavonoids (157 ± 1.3 μg·mL^−1^, 862 ± 1.5 μg·mL^−1^, and 303 ± 1.0 μg·mL^−1^, respectively). MA1 and MA4 associations with maize roots were assessed as root colonization percentages. Out of six endophytic fungal isolates, MA1 and MA4 ably showed colonization potential with maize roots (63% and 68%, respectively) (Figure 1B).

The molecular characterization of ACC deaminase-producing fungal endophytes was performed through phylogenetic analysis. The genomic DNA isolated from fungal mycelia was used to identify ACC deaminase-producing fungal endophytes based on 18S rDNA sequencing. The sequence homology of MA1 appeared with *Aspergillus nomiae* (97%) with NCBI accession no. OQ991163 and MA4 with *Aspergillus fumigatus* (98%) with NCBI accession no. OQ991162 (Figure S1A–C).

### 3.2. Assessment of ACC Deaminase-Producing Fungal Endophytes Using the Ethylene Response 1 Mutant Bioassay

Mutations in *ethylene overproducer1 (eto1)*, which is a negative regulator of an *ACS5* gene (encoding ACC synthase, the first step in the pathway), induced ethylene overproduction by synthesizing the ACC precursor of ethylene. Ethylene overproduction in the *eto1* mutant is limited mainly to etiolated seedlings only, which leads to a phenotype called the triple response: shortened and swollen hypocotyls, exaggerated apical hooks, and short roots. The triple response phenotype in the *ethylene overproducer1 (eto1)* mutant is because it generated five–ten fold higher ethylene levels than the wild type [61,62].

By taking advantage of the triple response phenotype of the etiolated *eto1* mutant in the present study, the growth-modulating potential through ET metabolism (catabolizing the ACC to the ketobutyrate and ammonia instead of ethylene) by ACC deaminase enzyme-producing fungal endophytes, etiolated Arabidopsis seedling bioassays were carried out by in vitro growing the *ET-overproducer (eto1)* mutant and Col-0 wild type seedling.

Out of six endophytes, MA1 and MA4 triggered restoration of ET-induced phenotypic abnormalities and the triple response morphology of etiolated *etol* seedlings compared with the Col-0 wild-type seedlings (Figure 1C,D). Etiolated hypocotyl growth dynamics, in terms of growth characteristic and root kinetics, were computed for etiolated *eto1* seedlings with MA1 and MA4, both individually and in combination, as well, revealing a reversion in total hypocotyl length the same as wild-type Col-0 (Figure 1C,D) due to the effect in which ACC produced by the etiolated *etol* seedlings was converted by the ACC deaminase enzyme activity of MA1 and MA4 fungal isolates and converted to a-ketobutyrate and ammonia, thus reducing the overproduction of ethylene to its optimal level. These observations are corroborated by the previous findings and the facts that, due to the presence of the ACC deaminase gene, it was previously known that several fungal species had the ACC deaminase ability to cleave the ET precursor ACC into a-ketobutyrate and ammonia, for example, the genomes of several *Penicillium* and *Trichoderma* species suggested that the fungus’ ACC deaminase controlled ET synthesis, which might be related to the plant’s resistance to various biotic and abiotic stimuli [63]. Until so far, it was unknown how ACC deaminase-producing fungal endophytes affect maize’s ability to withstand WS.

Recent research has shown that ACC deaminase is not secreted, but rather found in the cytoplasm of bacteria, while in plants, the excess ACC is synthesized inside the plant roots under waterlogged circumstances, and it is discharged from the plant tissues. Exuded ACC is absorbed and cleaved by bacteria that produce ACC deaminase and are linked to plant roots [64]. For researchers, in comparison to ACC deaminase enzyme bacteria, ACC deaminase enzyme-producing fungal endophytes seem to be more efficient as fungal hyphae penetrate within plant tissues (intercellular, as well as intracellular), thereby efficiently modulating ethylene production through ACC deaminase activity. Therefore, the current research was performed to evaluate the potential of ACC deaminase-producing fungal endophytes for alleviation of ethylene (ET)-induced stress in maize under waterlogging conditions.

There have only been a few findings before regarding ACC deaminase-producing fungal endophytes connected with plant abiotic stress tolerance. Recently it is investigated thet the beneficial influence of the ACC deaminase-producing fungal endophyte *Trichoderma asperellum* (MAP1) on plant–microbe interaction for enhancing WS tolerance in wheat by modulating ET and polyamines production [18]. *T. asperellum* T203 produced ACC deaminase, which controlled endogenous ACC levels in plant roots, stimulated root elongation, and enhanced plant tolerance to abiotic stress [27].

### 3.3. ACC Deaminase Enzyme Activity and Transcript Abundance in MA1 and MA4

**Figure 2:**
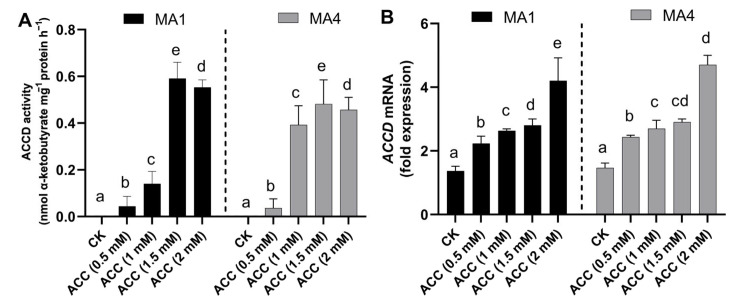
Quantification of ACC deaminase enzyme activity and ACC deaminase gene expression in MA1 and MA4 fungal biomass. (**A**) ACC deaminase enzyme activity, (**B**) ACC deaminase gene expression. Quantitative data show means ± SE, with various letters indicating significant difference (*p* ≤ 0.05).

MA1 and MA4 isolates ably produced higher biomass on the ACC-supplemented Czapek media, along with elevated potential for the ACC deaminase activity, which was recorded up to 0.58 µmol and 0.46 µmol of α-ketobutyrate mg^−1^ h^−1^, respectively, in Czapek media with ACC supplementation (2 mM), in a dose-dependent manner (Figure 2A).

MA1- and MA4-harboring ACC deaminase enzyme genes *(AFUA_2G01030* and *XM_015553204,* respectively) showed the induction of gene expression in an ACC-dependent manner. ACC deaminase enzyme gene expression analysis showed an upregulation of the ACC deaminase enzyme transcripts of MA1 (1.85-fold) and MA4 (2.5-fold) in comparison to the control, as shown in Figure 2B.

The ACC deaminase enzyme is thought to control ethylene synthesis because the ET precursor ACC can be catabolized to a-ketobutyrate and ammonia by its activity [29]. Numerous microorganisms have been found to produce the ACC deaminase enzyme, but among eukaryotes, some fungi, including a few species of yeast, like *Hansenula saturnus* and *Issatchenkia occidentalis*; other fungi, like *Penicillium citrinum* and *Trichoderma asperellum*; and a stramnopile called *Phytophthora sojae,* are particularly well known for producing it [27,65,66,67,68].

### 3.4. Effect of ACC Deaminase-Producing MA1 and MA4 on Maize Plants under WS

#### 3.4.1. Phenotypic Response of Maize under WS upon MA1 and MA4 Inoculation

**Figure 3:**
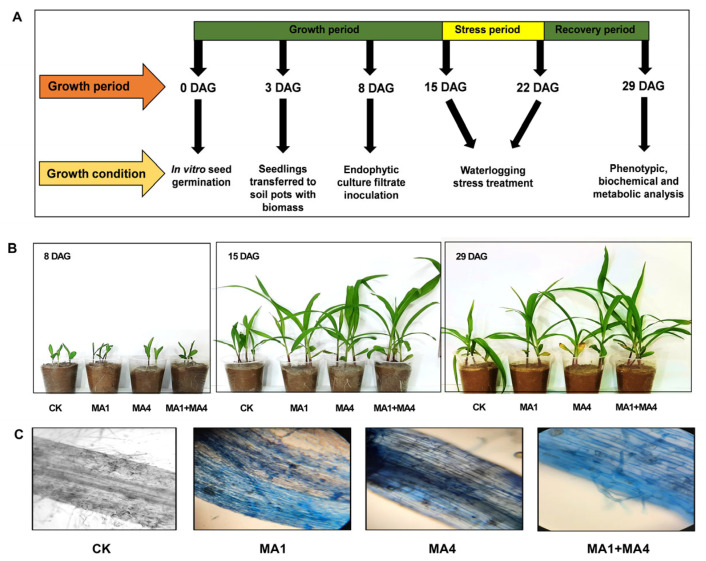
Phenotypic analysis of maize plants associated with MP1 and MA4 endophytic fungi. (**A**) Schematic representation of the maize bioassay showing experimental plan, with arrows indicating the timepoint of experimental activities. (**B**) Phenotypes of 8-day-old maize seedlings inoculated with fungal culture filtrate and biomass. The 15-day-old plants exposed to WS (8 days) with subsequent recovery period (8 days). (**C**) Microscopic visuals maize root colonized by MA1 and MA4 fungal associates.

In the present study, for the first time it is explored how the potential of ACC deaminase-producing fungal endophytes (*Aspergillus nomiae* and *Aspergillus fumigatus)* has affected the maize’s ability to withstand WS. To this end, phenotypic analysis was performed at four different time points (Figure 3A). Observation showed that the overall growth response was increased in MA1- and MA4-inoculated maize seedlings under WS in comparison to non-inoculated seedlings, following the growth phenotype (Figure 3B). The success of the plant–microbe interaction in the root tissue of the maize plants under observation was confirmed by lactophenol cotton blue staining, which showed that maize plant roots inoculated with MA1 and MA4 were ably colonized by endophytic fungi (Figure 3C).

#### 3.4.2. Growth Kinetics and Photosynthetic Activity of Maize Inoculated with MA1 and MA4 under WS

The growth kinetics in terms of shoot length, root length, and dry weight of the whole plant are presented in Table 3. A reduction in shoot length, root length, shoot dry weight, and root dry weight was found in WS-treated plants. However, MA1 and MA4 individual inoculations increased the WS tolerance of maize plants, as indicated by increased values of the shoot length, root length, and dry weight in comparison to the non-inoculated plants under WS stress. Moreover, combined inoculation of MA1 and MA4 significantly (*p ≤* 0.05) elevated the shoot length (70%), root length (83%), shoot dry weight (415%), and root dry weight (344%) in comparison to the non-inoculated plants under WS stress (Table 3).

The chlorophyll a, chlorophyll b, total chlorophyll, and carotenoid content showed a reduction in WS-treated plants compared with the control. However, MA1 and MA4 in individual inoculations significantly (*p ≤* 0.05) elevated the WS tolerance of maize plants compared with controls. Similarly, MA1 and MA4 co-inoculated plants further showed a significant (*p ≤* 0.05) increase in chlorophyll a (110%), chlorophyll b (90%), total chlorophyll (79%), and carotenoid content (257%) in comparison to the non-inoculated plants under WS stress (Table 3).

Waterlogging caused a lack of soil oxygen, which inhibited the uptake and transportation of nutrients and decreased grain yield [69]. In previous research, limited leaf photosynthesis and RuBPcase enzyme activity led to a decrease in the photosynthetic product [70,71], which in turn affected nutrient uptake and, consequently, decreased yields. Waterlogging may affect 12% of the world’s agricultural land, potentially leading to a 20% yield loss, and waterlogging significantly decreased the maize grain yield by shortening and widening the ear and lengthening the bald tip [3]. The performance of plants in response to waterlogging is strongly influenced by the stage of plant development, the depth of the water, and the frequency of waterlogging events. In the early growing stage of maize, waterlogging for 4 days reduced seedling growth [72], plant height, internode length, and stem diameter, and, to varying degrees, decreased the leaf area index (LAI) and CCI of maize leaves [73,74]. Additionally, early-growth-stage LAI was more significantly limited by waterlogging than later-growth-stage LAI, which further impacted maize photosynthesis and plant growth [70]. Under WS at the early growth stage, the maize yield was reduced by 58.8% to 69.8% [75]. In addition to this, WS tolerance has been modulated to increase by adopting various strategies, such as supplementing various exogenous chemical inducers, for example, urea by [73], and exogenous hormone 6-benzyladenine (6-BA) by [74], or by exploiting agriculture practices, for example, the making of ridge tillage for maize growth in the field, as reported by [76].

**Figure 4:**
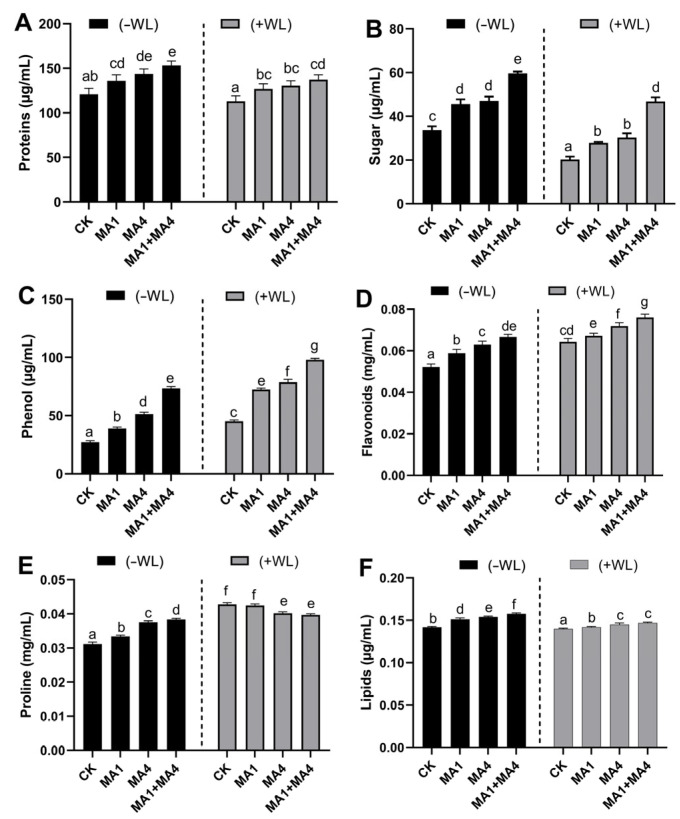
Growth-promoting metabolites in maize plants under WS, inoculated with MA1 and MA4. (**A**) Total protein content; (**B**) Total soluble sugars; (**C**) Total lipids; (**D**) Proline content; (**E**) Total phenolics; (**F**) Total flavonoids. Quantitative data represent means ± SE, with various letters indicating significant difference (*p* ≤ 0.05).

Prior research examined how maize plants responded physiologically and agronomically to waterlogging at specific growth stages and for varying lengths of time [75]. Researchers have recently concentrated on using endophytic fungi, like *Trichoderma*, which are identified as ACC deaminase- and IAA-producing fungi, to improve WS tolerance in plants [77]. Inducing WS tolerance in wheat at early growth stages by modulating ethylene and polyamine production under WS has recently been attributed to ACC deaminase-producing *Trichoderma asperllum* [18]. However, maize under WS tolerance induction has not been explored yet in the context of growth-promoting, ACC deaminase-producing fungal endophytes. The results of our study are the first to show how waterlogging, which lowers the amount of chlorophyll in maize plants, significantly lowers photosynthetic activity. The yield of waterlogged maize was significantly increased because of the inoculation of MA1 and MA4 endophytic fungi, which helped improve leaf photosynthesis in waterlogged maize by increasing chlorophyll content (Figure 4). It was primarily attributed to the capacity of endophytic fungi to take up nutrients from the soil, which enhanced the root system’s habitational environment and increased the root vitality and absorption capability [17]. Additionally, fungal colonization of root and stem tissue could enhance nutrient uptake and circulation to the plant’s photosynthetic tissues (leaf). This enhanced nutrient transportation and assimilation helped waterlogged maize produce more fresh weight and dry weight, shoot and root length, and overall biomass.

#### 3.4.3. Growth-Related Metabolites in Maize Inoculated with MA1 and MA4 under WS

The effect of MA1 and MA4 endophytes on growth-related metabolites of maize under WS was also assessed in comparison to the control. Both MA1 and MA4 individual inoculation significantly (*p ≤* 0.05) elevated the total soluble sugars, proteins, lipids, phenolics, and flavonoids in maize under WS compared to non-inoculated control plants under stress, while proline content was reduced, indicating the lower degradation of proteins in maize inoculated with MA1 and MA4 endophytes under WS (Figure 4).

Moreover, MA1 and MA4 co-inoculation further enhanced the production of total soluble protein (21%), total phenolics (113%), total soluble sugars (161%), total flavonoids (24%), and lipid content (5%) in maize plants under WS, compared with the non-inoculated maize plants under WS, while proline content was further reduced (17%) in maize co-inoculated with MA1 and MA4 endophytes under WS, compared with the non-inoculated maize plants (Figure 4).

#### 3.4.4. Endogenous ROS Production in Maize Plants under WS, Inoculated with MA1 and MA4

**Figure 5:**
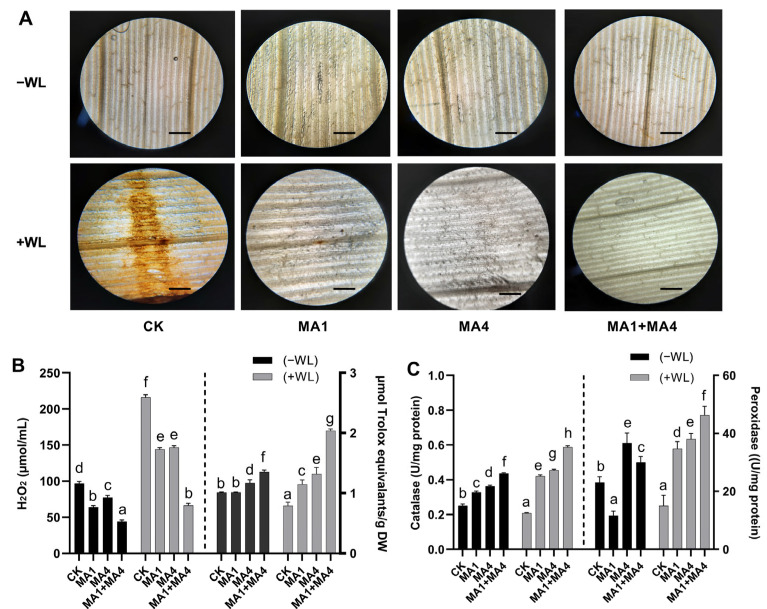
Antioxidant potential in maize plants under WS inoculated with MA1 and MA4. (**A**) DAB staining using 1 cm long segments of 3rd leaf from 29-day-old seedlings with and without endophytic fungal inoculation. Scale bar: 50 µm. (**B**) H_2_O_2_ production (**left** penal), DPPH activity (%) (**right** penal), (**C**) Catalase activity (**left** penal), and Peroxidase activity (**right** penal). Quantitative data represent means ± SE, with various letters indicating significant difference (*p* ≤ 0.05).

Endogenous H_2_O_2_ content was visualized as brown precipitates in 1 cm long segments of the 4th leaf from 27-day-old seedlings using the DAB staining method in Figure 5. The WS had a significant impact on the accumulation of reactive oxygen species, such as H_2_O_2_. In contrast, MA1 and MA4 inoculation reduced H_2_O_2_ production (69%) by lowering reactive oxygen species production and causing slight brown staining in the segments of leaf compared with the non-inoculated maize plants under WS (Figure 5A,B).

ROS, such as H_2_O_2_ and O_2_, are formed in peroxisomes, mitochondria, and chloroplasts as a result of homeostatic activities, as well as in reaction to stressors. Plant exposure to pathogen infection, drought, pollution, and waterlogging stressors have all been linked to an increase in ROS generation. They harm plant cells by producing oxidative damage to lipids, proteins, and nucleic acids. The development of programmed cell death-mediated aerenchyma by ROS as a method to cope with hypoxic stress is a well-studied phenomenon. Plants can limit the amount of oxidative damage based on their response by activating antioxidant enzymatic activities and producing antioxidants, such as glutathione, ascorbic acid, and polyphenols [7].

#### 3.4.5. Antioxidant Potential in Maize Inoculated with MA1 and MA4 under WS

Total antioxidants capacity (DPPH activity%) and antioxidant enzyme activities (CAT and POD) were also affected upon MA1 and MA4 inoculation in WS-exposed maize plants. Under WS, MA1 and MA4 individual inoculation significantly (*p ≤* 0.05) induced the DPPH activity and antioxidant enzyme activities of CAT and POD in non-inoculated maize plants. However, MA1 and MA4 co-inoculation resulted in a further increase in the DPPH activity (150%) and antioxidant enzyme activities of CAT (200%) and POD (207%) in non-inoculated maize plants exposed to WS (Figure 5C).

The quick increase in ethylene caused by NO overproduction governs the rise in ROS in hypoxic maize roots. An increase in ROS triggered cell death largely in the quiescent center (QC), limiting root development. As one of the early movers in the stress response, a mechanism for NO scavenging or ethylene bio reduction would be a crucial aspect in avoiding additional ROS-oxidative damage and excess PCD [7].

According to the current findings, maize plants’ capacity to scavenge ROS was improved by increasing the antioxidant, and waterlogging-sensitive lines induced high antioxidant enzyme activities in the presence of endophytic fungi (MA1 and MA4), which allowed them to fend off oxidative damage brought on by waterlogging.

Absent the inoculation of MA1 and MA4 endophytic fungi, the hypoxic stress induced by waterlogging imposed ROS overproduction, leading to the breakdown of soluble protein (as indicated by higher proline content) and the sugar and lipid content, which, in turn, caused the disorder of the ROS scavenging system and the acceleration of leaf senescence. Cells were guarded against photooxidative damage by the ROS scavenging system. By using antioxidant enzyme systems, crops could maintain the dynamic equilibrium of ROS, reducing membrane peroxidation and the severity of oxidative damage brought on by abiotic stresses (low temperature, waterlogging, drought, etc.) [6]. According to [78], SOD, POD, and CAT were the main antioxidant enzymes found in plant cells, and their activities showed how well plants could delay aging. An essential indicator, DAB staining, revealed the synthesis and buildup of H_2_O_2_. Our study demonstrated that after the waterlogging condition, the CAT and POD activities were reduced, while DAB staining (H_2_O_2_ production) increased, indicating the harmful influence of WS on antioxidant enzyme systems, resulting in an imbalance of ROS and membrane deterioration, and hastening the aging of leaves. Crops’ ability to reduce oxidative damage brought on by waterlogging can be improved by increasing the antioxidant enzyme activities [13].

However, the inoculation of MA1 and MA4 endophytic fungi assisted in improving soil root activity and postponing leaf senescence, supplying the aboveground organ with an adequate supply of nutrients, and thereby enhancing antioxidant activities. The inoculation of MA1 and MA4 endophytic fungi effectively increased the ROS-scavenging ability of maize under WS by elevating the antioxidant enzyme activities, countering oxidative stress, and alleviating waterlogging damage on the cell membrane system, resulting in a comparatively stable biological state. Current outcomes also exposed that inoculation of MA1 and MA4 endophytic fungi was advantageous to improve the POD and CAT activities and reduce ROS production. These findings demonstrated that the inoculation of MA1 and MA4 endophytic fungi could successfully lessen the harm caused by waterlogging to leaves, promptly remove reactive oxygen species within a certain range, maintain the function of leaves, and thereby improve the growth and yield of maize under WS.

#### 3.4.6. Hormonal Contents in Maize Inoculated with MA1 and MA4 under WS

**Figure 6:**
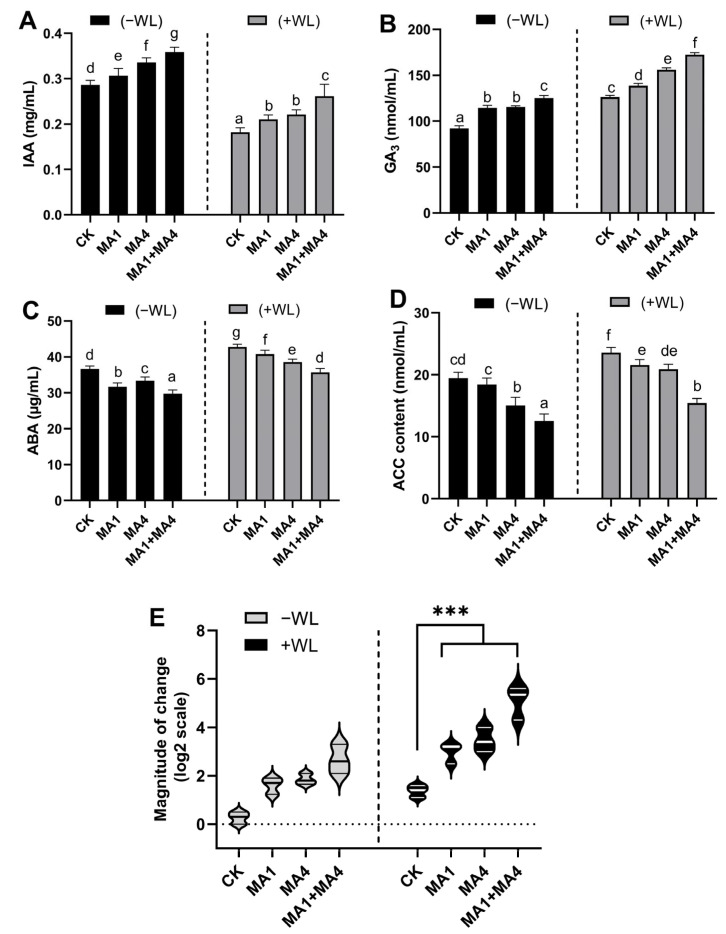
Growth- and stress-related hormonal content in maize plants under WS inoculated with MA1 and MA4. (**A**) IAA content; (**B**) GA_3_ content; (**C**) ABA content; (**D**) ACC content; and (**E**) Ethylene-responsive/waterlogging-induced marker gene expression analysis. Quantitative data represent means ± SE, with various letters indicating significant difference (*p* ≤ 0.05). The *** indicate the significant difference at *p* ≤ 0.05.

Endogenous IAA, GA_3_, ACC, and ABA levels were altered in response to individual and co-inoculation of MA1 and MA4 isolates in WS plants compared to non-inoculated control plants. Co-inoculation of MA1 and MA4 significantly (*p* ≤ 0.05) elevated the synthesis of GA_3_ (38%) and IAA (70%) (growth-related hormones) in maize plants under WS, compared to non-inoculated control plants under WS stress (Figure 6A,B).

Contrary to this, the endogenous ABA (16%) and ACC (34%), precursors of ethylene (stress-related hormones) significantly (*p* ≤ 0.05) reduced in response to MA1 and MA4 co-inoculation to the maize plants at 29 DAG, compared with the non-inoculated control plants under WS stress (Figure 6C,D).

The advantageous effects of endophytic fungi on plant growth and enhanced resistance to both biotic and abiotic stresses are well documented [21,37,79]. Nevertheless, the molecular basis of plant growth promotion by ACC deaminase-producing fungal endophytes is still unclear.

The growth-promoting action of *Trichoderma atroviride* on tomato seedlings has recently been linked to a reduction in ethylene production caused by microbial degradation of indole acetic acid in the rhizosphere and/or ACC deaminase enzyme activity present in the microorganism [80]. Ref. [77] demonstrated the importance of auxin signaling in the enhancement of plant development by *Trichoderma virens* in Arabidopsis.

In this work, ACC deaminase enzyme-producing fungal endophytes were isolated and investigated for their potential in the growth promotion of maize under WS. Using a genetic and biochemical approach, we explored how ACC deaminase enzyme is involved in the induction of plant growth promotion in maize under WS.

By modifying the size of guard cells, ABA plays a key role in controlling stomata, which, in turn, controls the water potential in plants. As a result, ABA is regarded as a crucial hormone in responses to water stress [81].

Previously, it was known that upon waterlogging, the ACC and ethylene may act as signaling molecules; however, overproduction and amplification of ethylene are stressful for plant physiological function, while ACC deaminase enzyme-producing fungi modulate ethylene levels and mitigate the adverse effects on plants in various stresses, such as flooding and drought. Nevertheless, further research is required to explore the role of some fungi, which utilized the ACC (ethylene precursor) as a carbon source for modulating plant growth under stress including waterlogging.

Researchers have previously demonstrated that ABA accumulation in the plant’s above-ground tissues under waterlogging was accelerated, with less water lost through transpiration and improved plant resistance to waterlogging through ABA-induced H_2_O_2_ accumulation and stomatal closure. The antioxidant defense system was triggered, and stomata closed because of the elevated ABA levels in *RAP2.6L*-overexpressing plants, which ultimately alleviated the oxidative stress, delayed senescence, and ably induced tolerance for waterlogging stress [11]. In wheat and other crops, waterlogging has been shown to significantly increase the amount of ABA [82]. In comparison to waterlogging treatment, ref. [83] discovered that adding exogenous ABA during waterlogging significantly increased soybean survival. Similarly, pretreatment with exogenous ABA had positive effects on the relative growth rate and chlorophyll content in rice plants under submersion [84].

Furthermore, waterlogging causes ET accumulation in the lower stem, which, in turn, reduces the ABA concentrations in the stem and AR primordia, suggesting that ABA (negatively) and ET (positively) are involved in the formation of adventitious roots primordia under waterlogging. Waterlogging-induced ARs were significantly inhibited by ABA treatment, but AR production was increased by an ABA inhibitor (Fluridone) [85].

These findings demonstrated that ABA negatively regulated the development of adventitious roots primordia under waterlogging, in contrast to ET. After waterlogging for 5 and 10 days, [86] measured the plant hormone content in soybeans with significant reduction in ABA content. The difference in ABA content between sensitive and waterlogging-resistant lines suggests the negative correlation between ABA and waterlogging tolerance when it comes to crosstalk with ET. Additionally, waterlogging in deepwater rice cultivars increased shoot elongation in part by decreasing endogenous ABA content and subsequently raising the GA concentration [13]. The present research also exposed the modulatory role of MA1 and MA4 for growth-promoting and stress-assuaging plant hormones. MA1 and MA4-induced reduction in ABA and ACC content might be corroborated by the WL-stress tolerance in maize plants, with a higher level of GA_3_ possibly contributing further to growth promotion under WL conditions.

#### 3.4.7. Ethylene-Responsive gene Expression in Maize Plants under WS, Inoculated with MA1 and MA4

Fungal endophytes with the ACC deaminase gene may degrade the ET precursor ACC to a-ketobutyrate and ammonia, as reported by [29]. ET is the smallest gaseous phytohormone that influences the JA and SA pathways that modulate responses for seed germination, fruit ripening, plant senescence, and plant immunity [30]. ET is generated in response to a variety of environmental biotic and abiotic stimuli, implying that it serves as a link between environmental change and developmental adaptability.

Previously, group VII ethylene response factors (ERFVIIs) have been directly known to play important roles in ethylene signaling and plant tolerance responses to waterlogging flooding in maize, as reported earlier, and the expression of *ZmEREB180* has already been known to induce upon waterlogging in maize [87]. By taking advantage of expression of *ZmEREB180*, waterlogging-induced marker gene, in the present study, the transcript abundance of the ethylene-responsive gene *ZmEREB180* was evaluated by RT-qPCR analysis. The expression of *ZmEREB180* specifically responded to waterlogging and was optimally expressed due to the moderate production of ACC, and exhibited the reduction by >fourfold in MA1- and MA4-inoculated maize plants under WS compared with the non-inoculated control, indicating the effect of ACC deaminase producing an endophytic association in modulating the ethylene-responsive *ZmEREB180* gene expression, as well (Figure 6E).

Multiple functions of ET as a signaling molecule have resulted in several biochemical linkages between ET and growth. Submergence, heat, shade, heavy metal, high salt exposure, limited food availability, and water scarcity are all abiotic stressors that cause ET synthesis [88]. The role of ET in flood-induced hyponastic leaf movement is well recognized, since *Arabidopsis thaliana aco5* mutants exhibit diminished leaf hyponastic responses to waterlogging [89]. Transcriptomic alterations have been found in wheat roots that have been treated with *T. harzianum*, either alone or in conjunction with varying doses of calcium nitrate [Ca(NO_3_)_2_] as a nitrogen (N) source. The Molecular and physiological characterization of the primary candidate genes implicated in plant waterlogging tolerance has been prioritized to better understand plant adaptation to waterlogging and stress tolerance response due to ET signaling.

The *miR159* expression was induced in the roots of maize under waterlogging, which initiated the mRNA-silencing of *GAMYBs* including *MYB33,* and *MYB101*, thus inhibiting the primary root growth by modulating the responses related to ABA and GA signaling pathways. The *miR166* and *miRNA167* were upregulated in response to short-term and long-term waterlogging in maize roots, which cause downregulation of its target, thus modulating ABA, GA, and auxin signaling pathways to optimize the lateral and adventitious rooting response. Both *miR393* and *miR164* were shown to be upregulated in waterlogged and flooded maize, suggesting that they may have a comparable effect on root development control in response to waterlogging by altering the auxin pathway [11].

During floods, ethylene signaling cause morphological and metabolic changes in plants. The *miR172* targets *APETALA2/Ethylene Responsive Element,* and long-term waterlogging in maize roots decreases *miR172* expression, promoting main root development. Flood-induced ethylene signaling affects root architecture, water transport, energy metabolism, and programmed cell death [12].

ET-response factors from Group VII (ERF-VIIs) have a significant impact on both ET signal transduction and plant responses to waterlogging [90]. The ERF-VII family member gene *ZmEREB180* in maize has been shown to favorably regulate the establishment and proliferation of adventitious roots and ROS production. *ZmEREB180* in maize triggered the survival rate under extended WS [87].

Following the previous report, the present study also emphasized the positive role of *ZmEREB180* in maize under WS controlled by MA1 and MA4 endophytic fungi, which eventually induced WS tolerance by optimizing phytohormone (ethylene) synthesis and ROS levels to reduce hypoxia-induced oxidative damage.

#### 3.4.8. Root and Stem Anatomical Features in Maize Plants under WS, Inoculated with MA1 and MA4

As reported earlier [91], hypoxia induced by the WS altered the morphological and anatomical structure of the maize plant root and stem. Aquatic adventitious root formation is considered an important adaptation of maize plants under waterlogging and flooding stress. Maize can produce adventitious roots that contain aerenchyma, a highly porous tissue type that facilitates O_2_ diffusion from the shoot into the root, improves the internal aeration of the plant, and allows energy-dependent root functions such as water and nutrient uptake to survive under waterlogging and flooding stress.

Exploring the effect of MA1 and MA4 on maize adaptability to waterlogging, the adventitious root formation, lysigenous aerenchyma formation, and the ratio of cortex-to-stele within the maize roots were evaluated phenotypically and microscopically.

**Figure 7:**
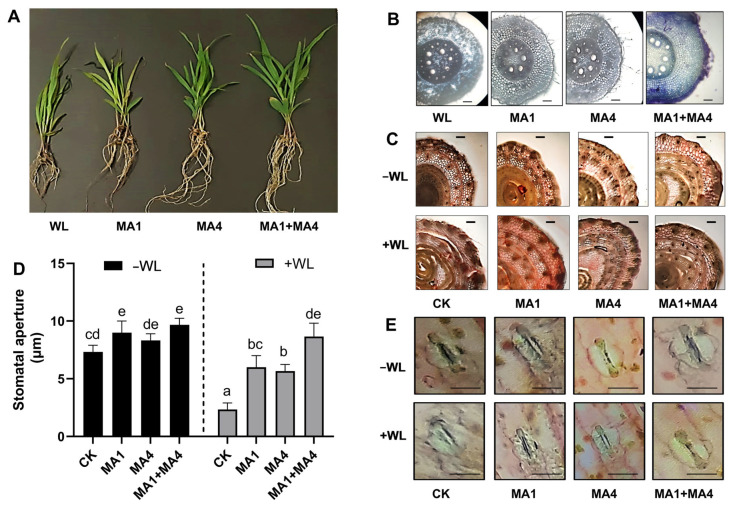
Root, stem, and stomatal anatomy in maize plants under WS inoculated with MA1 and MA4. (**A**) Phenotype of maize plants; (**B**) Microscopic visuals of root anatomical features. Scale bar: 50 µm; (**C**) Stem anatomical features. Scale bar: 100 µm; (**D**) Stomatal aperture. Scale bar: 10 µm; (**E**) Stomatal anatomy. Quantitative data represent means ± SE, with various letters indicating significant difference (*p* ≤ 0.05).

Adventitious root formation was increased in MA1- and MA4-inoculated maize plants under WS compared with the control and non-inoculated plants (Figure 7A). The root and stem anatomy visualized by microscopy showed the typical features related to WS responses, such as fewer lysogenous spaces and reduced water-filled cells, smaller cell gaps, tight and round cells, and intact cortical cells without damage. (Figure 7B). The stem sections from maize in the absence of endophytic association under WL stress showed slightly deformed mesophyll cells, deteriorated epidermal and vascular bundle cells, as well as swollen metaxylem, phloem, pith, and cortical area (Figure 7C).

Several plant species under flooding, can grow adventitious roots on their submerged stems. These roots have aerenchyma, a highly porous tissue type that allows O_2_ to diffuse from the shoot into the root. This enhances the plant’s internal aeration, especially while sections of the shoot are still spreading above the floodwater surface, and permits energy-dependent root processes like water and nutrient absorption to continue. As a result, adventitious root production is regarded as a key adaptation of plants to floods, including maize [91].

Aerenchyma is made up of longitudinally linked gas pockets that aid in the diffusion of oxygen throughout plants. Lysigenous aerenchyma in roots is created by the formation of gas gaps as a result of the death and subsequent lysis of cortical cells [92]. It was previously established that a bigger stele with lower porosity than the cortex has a disadvantage in terms of oxygen transport within the root. These findings imply that in addition to aerenchyma, root thickness and the ratio of cortex-to-stele within roots are important factors in determining plant adaptation to waterlogging. Thick root diameter is related to the bigger cortex and aerenchyma portions anatomically. Under oxygen-deficient circumstances, the quantity of aerenchyma in maize rises [92].

Rice root aerenchyma is generated by WS due to the production of *ACS1* and *ACO5*, which promotes ET synthesis. At the same time, ET promotes cortical cell death, which is mediated by ROS and results in the development of aerenchyma. The ET accumulates in roots during waterlogging due to its continuing production and slow water diffusion rate. ET promotes programmed cell death during lysogenic aerenchyma formation [93]. The accumulation of ET in maize, rice, and wheat stimulates the formation of lysosomal aerenchyma [92,94]. Ref. [95] found that H_2_O_2_ pretreatment improved soybean WL tolerance by increasing the net photosynthetic rate and antioxidant system activity under WL, while concurrently lowering ROS levels and the severity of biological membrane destruction. 

The current study found that when maize stem was exposed to hypoxia caused by WS, it experienced cortical cell death and lysogeny, which is mediated by ROS and results in the development of aerenchyma. Yet, similar detrimental traits were absent in the stem architecture of maize injected with endophytic fungus (MA1 and MA4), under WS. This might be attributed to the ACC deaminase activity of the endophytic fungi colonizing the maize plants under WL stress, which leads to decreased ACC content generation and reduced conversion to ET, resulting in reduced ROS production and cell death.

Hypoxia, drought, and nutritional shortages all cause root cortical aerenchyma (RCA). Prior studies found that RCA development lowers root tissue respiration and nutrient content. By lowering the metabolic costs of soil exploration in maize, RCA development is a helpful adaptation to poor phosphate, nitrogen, and potassium availability [96]. Aerenchyma production in maize allows oxygen to travel from shoots to roots in waterlogged soil, allowing plants to survive under waterlogging-induced hypoxic stress [92]. Under aerobic conditions, ethylene production typically precedes the formation of aerenchyma in maize roots, and a low level of ACC production is probably the rate-limiting step for ethylene biosynthesis in these aerobic roots. Conversely, the levels of ethylene synthesis in maize roots were related to a more significant rise in ethylene production immediately following the commencement of low-oxygen circumstances [94]. However, moderate ethylene production is essential for plants experiencing water stress because excessive ethylene production can result in an accumulation of ROS that can cause cell death.

#### 3.4.9. Stomatal Regulation in Maize Plants under WS, Inoculated with MA1 and MA4

The influence of MA1 and MA4 isolates on the stomatal anatomy and stomatal aperture of maize under WS, was also explored. Individual and combination inoculation of MA1 and MA4 isolates resulted in a substantial (*p ≤* 0.05) increase in stomatal aperture size under normal and WL stress conditions compared to control. In comparison to controls, maize with combination inoculation of MA1 and MA4 normal and WL stress conditions showed the greatest increase in stomatal aperture. Although WL stress resulted in the greatest decrease in the stomatal aperture in maize when compared to a non-inoculated control (Figure 7D,E).

ABA has an important function in managing stomata, which in turn controls the water potential in plants, by altering the size of guard cells. As a result, ABA is recognized as a critical hormone in water stress reactions. Abscisic acid, a “negative regulator”, is also implicated in the creation of root aerenchyma when there is waterlogging because secondary aerenchyma formation needs a decrease in ABA production [97]. Endogenous ET production and aerenchyma cell development are both interconnected processes [98].

## 4. Conclusions

The present work exposed the potential of ACC deaminase-producing fungal endophytes (MA1 and MA4) to not only endogenously produce and secrete the growth-promoting regulator and metabolites, but also trigger the basal threshold of stress-alleviating and growth-promoting metabolites of host plant maize under waterlogging-induced hypoxia stress. In conclusion, the current study emphasizes the potential of MA1 and MA4 endophytic fungi in the growth promotion of maize by adequate production of IAA, GA_3_, phenols, and flavonoids, with increased chlorophyll content and stomatal conductance resulting in greater biomass. WS tolerance in maize plants was also induced due to the bio-reduction of ethylene to an optimal level through ACC deaminase activity of MA1 and MA4 endophytic fungi, as depicted in the graphical abstract. In addition, antioxidant activities of enzymatic and nonenzymatic entities, such as POD, and CAT in maize plants under WS were also enhanced and exponentiated by MA1 and MA4, leading to the reduction in ROS. MA1 and MA4 also positively affected the expression of *ZmEREB180* in maize for modulation in ET signal transduction and plant responses to waterlogging. The present study also revealed that MA1 and MA4 endophytic fungi could be employed as bioengineers for healthy maize crop production under WS, particularly in flood-prone regions, due to the ability for reshuffling the ET metabolism by exploiting their ACC deaminase enzyme activity and controlling the expression of *ZmEREB180* in maize under WS.

## Figures and Tables

**Table 1:** The physicochemical properties of the soil used to grow the wheat plants.

Characteristics	Values
Texture	Sandy-loam
Silt (%)	12.9
Sand (%)	75.8
Clay (%)	15.2
ECe (dS/m)	0.8
CEC (dS/cm)	4.6
pH	7.8
Chlorides (meq/L)	1.17
Bicarbonates (meq/L)	2.8
Carbonates (meq/L)	1.30
Organic Carbon (%)	4.21
Organic matter (%)	1.5

**Table 2:** *qPCR* primers used for *ZmEREB180* expression.

Gene	Forward/Reverse	Sequence of Primers
*ZmActin1*	Forward	*TACGAGATGCCTGATGGTCAGGTCA*
	Reverse	*TGGAGTTGTACGTGGCCTCATGGAC*
*ZmEREB180* (GRMZM2G018984)	Forward	*AGAGGAAGGAAGGGATCGC*
	Reverse	*GAGTCTTCGTCGCATCTCG*

**Table 3:** Growth kinetics and photosynthetic activity in maize plants under WS inoculated with MA1 and MA4.

Parameters	Treatments/ Conditions	CK	MA1	MA4	MA1 + MA4
Shoot length	−WL	21.37 ± 0.35 ^b^	27.5 ± 0.4 ^d^	30.5 ± 0.3 ^g^	34.53 ± 0.4 ^h^
	+WL	17.5 ± 0.29 ^a^	23.43 ± 0.29 ^c^	26.33 ± 0.29 ^d^	29.07 ± 0.29 ^f^
Root length	−WL	20.83 ± 0.76 ^c^	23.2 ± 0.26 ^e^	25.2 ± 0.3 ^f^	27.23 ± 0.25 ^g^
	+WL	12.27 ± 0.25 ^a^	19.2 ± 0.16 ^b^	20.2 ± 0.22 ^c^	22.03 ± 0.21 ^d^
Fresh weight	−WL	1.15 ± 0.04 ^d^	1.28 ± 0.01 ^e^	1.37 ± 0.05 ^f^	1.43 ± 0.02 ^f^
	+WL	0.19 ± 0.01 ^a^	0.93 ± 0.02 ^b^	0.9 ± 0.04 ^b^	1.05 ± 0.06 ^c^
Dry weight	−WL	0.14 ± 0.08 ^d^	0.43 ± 0.06 ^e^	0.36 ± 0.05 ^f^	0.73 ± 0.09 ^f^
	+WL	0.08 ± 0.01 ^a^	0.16 ± 0.03 ^b^	0.23 ± 0.02 ^b^	0.5 ± 0.11 ^c^
Chlorophyll a	−WL	4.47 ± 0.04 ^d^	5.07 ± 0.05 ^e^	5.24 ± 0.09 ^f^	6.35 ± 0.05 ^g^
	+WL	2.08 ± 0.1 ^a^	3.47 ± 0.09 ^b^	4.11 ± 0.11 ^c^	4.49 ± 0.08 ^d^
Chlorophyll b	−WL	1.39 ± 0.05 ^c^	1.59 ± 0.04 ^d^	2.52 ± 0.05 ^e^	2.9 ± 0.06 ^f^
	+WL	0.69 ± 0.03 ^a^	1.15 ± 0.04 ^b^	1.36 ± 0.04 ^c^	2.55 ± 0.03 ^e^
Total chlorophyll	−WL	8.87 ± 0.15 ^e^	9.97 ± 0.08 ^f^	10.54 ± 0.04 ^g^	12.23 ± 0.11 ^h^
	+WL	4.88 ± 0.03 ^a^	6.34 ± 0.15 ^b^	7.66 ± 0.11 ^c^	8.28 ± 0.24 ^d^
Carotenoids	−WL	1.39 ± 0.05 ^c^	1.59 ± 0.04 ^d^	2.52 ± 0.05 ^e^	2.9 ± 0.06 ^f^
	+WL	0.7 ± 0.03 ^a^	1.15 ± 0.04 ^b^	1.36 ± 0.04 ^c^	2.55 ± 0.03 ^e^

Note: Quantitative data represent means ± SE, with various letters indicating significant differences (*p* ≤ 0.05), according to Duncan’s Multiple Range test.

## Data Availability

The whole datasets presented in this article have been included in the manuscript and submitted to the NCBI repository. Requests for further information should be directed to MR at mamoona@awkum.edu.pk.

## Supplementary Materials

The following supporting information can be downloaded at: https://www.mdpi.com/article/10.3390/microorganisms11082025/s1, Figure S1: Genotyping of selected fungal isolates by ITS region amplification (A). Phylogenetic analysis for molecular identification of MA4 isolate (B). MA1 isolate (C).
