# Supplementary material for: Aspergillus nomiae and fumigatus Ameliorating the Hypoxic Stress Induced by Waterlogging through Ethylene Metabolism in Zea mays L
Source: Microorganisms. 2023 Aug 7;11(8):2025. doi: 10.3390/microorganisms11082025 (PMC10459883; doi:10.3390/microorganisms11082025)
Supplement: Supplementary file 1 [file microorganisms-11-02025-s001.zip › microorganisms-2489230-supplementary.pdf]

Aspergillus nomiae and *fumigatus* Ameliorating the Hypoxic Stress Induced by Waterlogging through Ethylene Metabolism in *Zea mays* L.

Khalil Ur Rahman [1], Kashmala Ali [2], Mamoona Rauf [2,*] and Muhammad Arif [1,*]

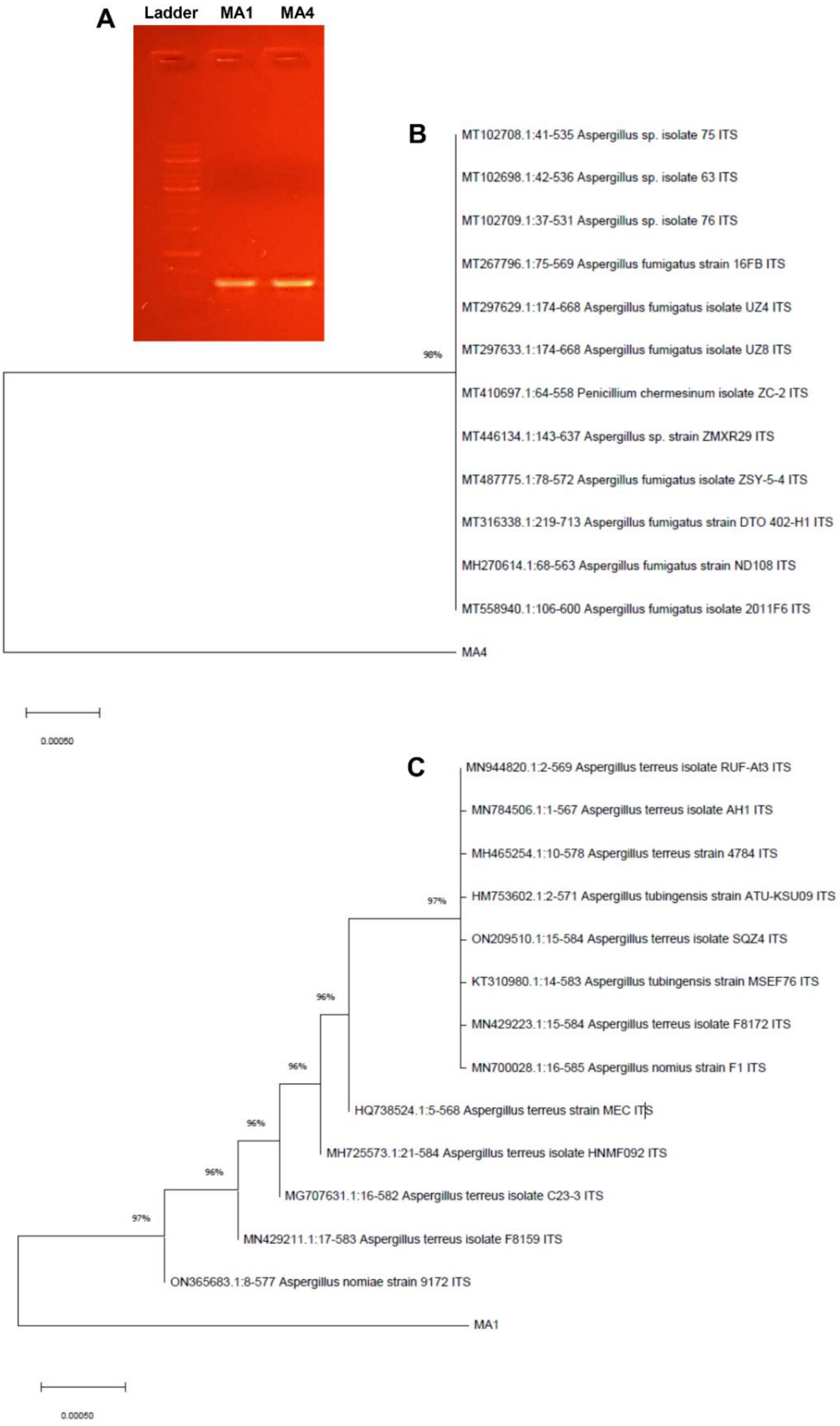

Figure S1. Genotyping of selected fungal isolates by ITS region amplification (A). Phylogenetic analysis for molecular identification of MA4 isolate (B). MA1 isolate (C).
